# Magnolia polyphenols attenuate oxidative and inflammatory responses in neurons and microglial cells

**DOI:** 10.1186/1742-2094-10-15

**Published:** 2013-01-29

**Authors:** Dennis Y Chuang, Ming-Huan Chan, Yijia Zong, Wenwen Sheng, Yan He, Jing Hua Jiang, Agnes Simonyi, Zezong Gu, Kevin L Fritsche, Jiankun Cui, James C Lee, William R Folk, Dennis B Lubahn, Albert Y Sun, Grace Y Sun

**Affiliations:** 1 Interdisciplinary Neuroscience Program, University of Missouri, Columbia, MO, USA; 2 Center of Translational Neuroscience, School of Medicine, University of Missouri, Columbia, MO, USA; 3 MU Center for Botanical Interaction Studies, Columbia, MO, USA; 4 Institute of Neuroscience, National Chengchi University, Taipei, Taiwan; 5 Department of Biochemistry, University of Missouri, Columbia, MO, USA; 6 Department of Biology, Program in Neuroscience, Syracuse University, Syracuse, NY, 13244, USA; 7 Department of Pathology and Anatomical Sciences, University of Missouri, Columbia, MO, USA; 8 Department of Animal Sciences, University of Missouri, Columbia, MO, USA; 9 Department of Biological Engineering, University of Missouri, Columbia, MO, USA

**Keywords:** ERK1/2, Honokiol, IFNγ, iNOS/NO, Inflammatory, Magnolol, Microglial cells, Oxidative, NADPH oxidase, Neurons

## Abstract

**Background:**

The bark of magnolia has been used in Oriental medicine to treat a variety of remedies, including some neurological disorders. Magnolol (Mag) and honokiol (Hon) are isomers of polyphenolic compounds from the bark of *Magnolia officinalis*, and have been identified as major active components exhibiting anti-oxidative, anti-inflammatory, and neuroprotective effects. In this study, we investigate the ability of these isomers to suppress oxidative stress in neurons stimulated by the ionotropic glutamate receptor agonist N-methyl-D-aspartate (NMDA) and oxidative and inflammatory responses in microglial cells activated by interferon-γ (IFNγ) and lipopolysaccharide (LPS). We also attempt to elucidate the mechanism and signaling pathways involved in cytokine-induced production of reactive oxygen species (ROS) in microglial cells.

**Methods:**

Dihydroethidium (DHE) was used to assay superoxide production in neurons, while CM-H2DCF-DA was used to test for ROS production in murine (BV-2) and rat (HAPI) immortalized microglial cells. NADPH oxidase inhibitors (for example, diphenyleneiodonium (DPI), AEBSF, and apocynin) and immunocytochemistry targeting p47phox and gp91phox were used to assess the involvement of NADPH oxidase. Western blotting was used to assess iNOS and ERK1/2 expression, and the Griess reaction protocol was employed to determine nitric oxide (NO) concentration.

**Results:**

Exposure of Hon and Mag (1–10 μM) to neurons for 24 h did not alter neuronal viability, but both compounds (10 μM) inhibited NMDA-stimulated superoxide production, a pathway known to involve NADPH oxidase. In microglial cells, Hon and Mag inhibited IFNγ±LPS-induced iNOS expression, NO, and ROS production. Studies with inhibitors and immunocytochemical assay further demonstrated the important role of IFNγ activating the NADPH oxidase through the p-ERK-dependent pathway. Hon and, to a lesser extent, Mag inhibited IFNγ-induced p-ERK1/2 and its downstream pathway for ROS and NO production.

**Conclusion:**

This study highlights the important role of NADPH oxidase in mediating oxidative stress in neurons and microglial cells and has unveiled the role of IFNγ in stimulating the MAPK/ERK1/2 signaling pathway for activation of NADPH oxidase in microglial cells. Hon and Mag offer anti-oxidative or anti-inflammatory effects, at least in part, through suppressing IFNγ-induced p-ERK1/2 and its downstream pathway.

## Background

The bark of the magnolia tree has been used in a number of traditional herbal medicinal preparations in Oriental countries for thousands of years [1]. *Magnolia officinalis* (China) and *Magnolia obovata* (Japan) contain a rich source of biologically active compounds, including alkaloids, coumarins, flavonoids, lignans, neolignans, and terpenoids [2,3]. According to *in vitro* and *in vivo* studies, and recent clinical trials, there is strong evidence that these constituents play an important role in the treatment of a plethora of ailments by reducing allergic and asthmatic reactions, and suppressing anxietic and angiogenic responses [4-9].

**Figure 1:**
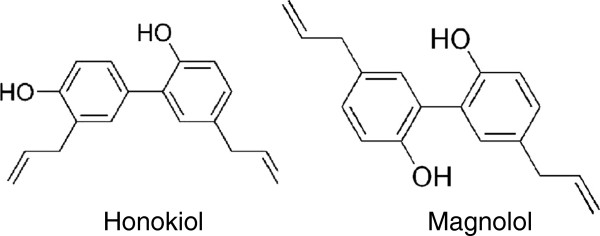
Structure of honokiol (Hon) and magnolol (Mag).

Magnolol (Mag) and honokiol (Hon) (Figure 1) are polyphenolic compounds from *Magnolia officinalis* belonging to the neolignan family. Recent evidence that these compounds exert beneficial effects in neurological disorders, such as anxiety, depression, stroke, Alzheimer’s disease, and Parkinson’s disease, has attracted great attention and further investigation of their molecular mechanism and specific targets [10-15]. Neuronal excitation due to stimulation by the ionotropic glutamate receptor agonists is known to elicit a rapid influx of calcium, which triggers downstream pathways leading to the production of reactive oxygen species (ROS) and mitochondrial dysfunction [16,17]. Understanding the underlying mechanism by which *Magnolia* compounds suppress neuronal excitotoxicity may help explain their ameliorating actions in disease models.

Studies with cell models have demonstrated anti-inflammatory effects of Hon and Mag in mitigating cytokine-induced nitric oxide (NO) production, expression of inducible nitric oxide synthase (iNOS), and generation of prostaglandins and leukotrienes [18-20]. This type of inflammatory response is important in microglial cells because their activation has been the basis of a number of neurodegenerative diseases. Although cytokines and LPS have been shown to activate microglial cells and induce ROS and NO production, mechanistic details within the signaling pathways leading to this type of oxidative and inflammatory responses have not been clearly elucidated.

In this study, we aim to test the ability for Hon and Mag to suppress oxidative and inflammatory responses in neurons and microglial cells. Studies with neurons were based on the excitotoxic model demonstrating the involvement of NADPH oxidase in NMDA-stimulated ROS production [16]. Studies with microglial cells demonstrated that NADPH oxidase was also involved in mediating cytokine and LPS-induced ROS production. In addition, our studies further unveiled the important role of the IFNγ-ERK1/2 signaling pathway for ROS production and the ability of Hon and Mag to suppress this pathway in microglial cells.

## Materials and methods

### Materials

Honokiol (lot number M8P0236) and magnolol (lot number M8F3374) (≥98% pure based on HPLC) were purchased from Nacalai Tesque, Inc. (Kyoto, Japan). These compounds were dissolved in dimethyl sulfoxide (DMSO) as stock solutions. DMEM, penicillin, streptomycin, 0.05% (w/v) trypsin/EDTA, and PBS were obtained from GIBCO (Gaithersburg, MD). Interferon-γ (IFNγ) was purchased from R&D Systems (Minneapolis, MN). Lipopolysaccharide (LPS) (rough strains) from *Escherichia coli* F583 (Rd mutant) and methylthiazolyldiphenyl-tetrazolium bromide (MTT) were obtained from Sigma-Aldrich (St. Louis, MO). The AlamarBlue™ kit was from Invitrogen (Carlsbad, CA). Fetal bovine serum was from Atlanta Biologicals (Lawrenceville, GA). Antibodies used for Western blotting include: goat anti-rabbit IgG-horseradish peroxidase, goat anti-mouse IgG-horseradish peroxidase, and iNOS polyclonal (Santa Cruz Biotechnology, Santa Cruz, CA); monoclonal anti-β-actin peroxidase (Sigma-Aldrich, St. Louis, MO); ERK1/2, phospho-ERK1/2, (Cell Signaling, Beverly, MA). Antibodies used for immunocytochemical staining include rabbit anti-p47phox antibodies (Calbiochem, Billerica, MA), mouse anti-gp91phox (Thermo Fisher, Waltham, MA), goat-anti-rabbit Alexa fluor 488 (Jackson Immunoresearch, West Grove, PA), and goat-anti-mouse Alexa fluor 549 (Jackson Immunoresearch, West Grove, PA).

For ROS detection, CM-H2DCFDA (DCF) was obtained from Invitrogen, Inc. (Carlsbad, CA), and dihydroethidium (DHE) from Sigma-Aldrich (St. Louis, MO). Inhibitors used in this study include: MEK inhibitor U0126 (Cell Signaling, Beverly, MA), 4-(2-aminoethyl)-benzenesulfonylfluoride (AEBSF, Calbiochem*,* San Diego, CA), diphenyleneiodonium (DPI) and apocynin (Sigma-Aldrich, St. Louis, MO).

### Cell culture

Preparations of primary cortical neuron cells involved pregnant E17 Sprague–Dawley rats (Harlan, IN, USA). All animal care and experimental protocols were carried out in accordance with National Institutes of Health (NIH) guidelines and with permission from the University of Missouri Animal Care and Use Committee (protocol #6728). Primary cortical neurons were prepared from the cerebral cortices of E17 Sprague–Dawley rat embryos as described [21]. Briefly, cerebral cortices were dissected and meninges removed. The tissues were suspended in 3 ml 0.05% (w/v) trypsin/EDTA and incubated for 30 min at 37°C. The cell suspension was triturated through a fine-burned-tip glass pipette until tissues were homogenized. The filtrate was centrifuged at 1000*g* for 1 min and resuspended in 10% FBS in DMEM containing 100 units/ml penicillin and streptomycin (100 μg/ml). Finally, cells were plated in 24-well plates for MTT analysis and 35 mm dishes for ROS detection. The plates were precoated with poly-L-lysine (Sigma-Aldrich, St. Louis, MO) the day before plating and incubated overnight. Four hours after plating, culture medium was completely changed to B27 supplemented Neurobasal medium containing 100 units/ml penicillin, 100 μg/ml streptomycin, and glutamine. Culture was maintained by changing 1/2 volume of B27 medium in each well every 4 days. Experiments were conducted 8 days after plating, to ensure adequate culture maturation.

The immortalized mouse BV-2 and rat HAPI microglial cells were cultured as described previously [22]. Briefly, cells were cultured in 75 cm^2^ flasks with DMEM (high glucose) supplemented with 10% FBS containing 100 units/ml penicillin and 100 μg/ml streptomycin, and maintained in a 5% CO_2_ incubator at 37°C. For subculture, cells were removed from the culture flask with a scraper, resuspended in the culture medium and subcultured in 12, 24, or 96-well plates for experiments.

### Assessing cell viability

Two assay protocols were used to assess neuron viability after exposure to Hon and Mag. Since mitochondrial dysfunction is an initial step in apoptotic pathways that lead to neuronal cell death, the MTT assay was used to determine mitochondrial dysfunction. In this assay, neurons were cultured in 24-well plates and treated with Hon or Mag (1 to 10 μM) at 37°C for 24 h. After treatment, culture medium was removed and 1 ml of MTT reagent (0.5 mg/ml) dissolved in serum-free DMEM was added to each well. The plates were incubated for 3 h at 37°C, and the formazan particles in each well were dissolved in 500 μl of DMSO. After shaking the plates at room temperature for 5 min, absorbance was read at 540 nm using a Synergy4 Plate Reader (BioTek Instruments, Inc., Fisher Scientific, St. Louis, MO, USA.).

AlamarBlue™ is a cell-permeable nonfluorescent dye, which can be converted into red fluorescence on reductive reactions within live cells. This assay was used to determine the extent of neuronal viability after exposure to Hon and Mag (1 to 10 μM). In this assay, neurons were cultured in 24-well plates and then treated with Hon or Mag (1 to 10 μM) at 37°C for 24 h. After treatments, 100 μl of AlamarBlue^TM^ was added to each well and neurons were further incubated at 37°C for 3 h. Absorbance was read at 570 nm using the Synergy4 Plate Reader (BioTek) with measurement at 600 nm as a reference.

### Measurement of superoxide in neurons and ROS production in microglial cells

Dihydroethidium (DHE) is oxidized by superoxide anions to produce fluorescent ethidium, which is intercalated into DNA, and can be quantified by summing the fluorescence within the cell [23]. This protocol has been successfully used to measure superoxide production in neurons [16,21]. Neurons were cultured on 35 mm dishes precoated with poly-L-lysine. Neurons were then treated with Hon or Mag (10 μM) for 30 min, and exposed to NMDA (100 μM) for 30 min in phenol red free Neurobasal medium with 0.5 mg/ml BSA. At 30 min prior to image acquisition, cells were loaded with DHE (10 μM) and incubated at 37°C. Fluorescence images were acquired using a Nikon TE-2000 U inverted microscope with a 20× NA 0.95 objective and a cooled charge-coupled device (CCD) camera controlled with a computer running MetaView imaging software (Universal Imaging, West Chester, PA). The fluorescence excitation source was controlled with a Uni-Blitz mechanical shutter. For image acquisition, a short exposure time (200 ms) and low-intensity excitation light were applied to minimize photobleaching. Digital images were analyzed using the MetaView software with automatic background subtraction. Threshold fluorescence levels were obtained for each image prior to the quantification. For each field, the total fluorescence was measured and expressed as the average fluorescence normalized by the total number of cells. For each treatment group, at least three random images from the same dish were captured and analyzed, and each treatment was repeated three times independently for statistical analysis.

For measurement of ROS production in microglial cells, we adopted a protocol using CM-H2DCF-DA, a compound that becomes fluorescent upon interacting with ROS including H_2_O_2_[24]. In this study, microglial cells were seeded in 96-well plates, and when they became 90% confluent, they were serum-starved for 4 h. Cells were treated with cytokines or LPS, or both, for different times and CM-H2DCF-DA (10 μM) was added 1 h before measurement. In some experiments, cells were pretreated with U0126 or Hon or Mag for 1 h prior to stimulating with IFNγ for 11 h, the CM-H2DCF-DA (10 μM) was then added and the cells were further incubated for 1 h. The fluorescent intensity of DCF was measured using the Synergy4 microplate reader with an excitation wavelength of 490 nm and an emission wavelength of 520 nm.

### Measurement of NO

Cells were serum-starved in phenol red free DMEM for 3 h, followed by pretreatment of compounds of interest for 1 h. Cells were then treated with IFNγ and LPS or IFNγ alone and incubated at 37°C for 16 h. Nitric oxide released from cells was converted to nitrite in the culture medium, which was determined using the Griess reagent protocol. Aliquots (50 μl) of culture medium were transferred to a 96-well plate and incubated with 50 μl of Reagent A (1% sulfanilamide in 5% phosphoric acid) per well for 10 min at room temperature, covered and in the dark. This was followed by incubation with 50 μl of reagent B (0.1%, w/v, N-1-napthylethylenediamine dihydrochloride, Sigma-Aldrich) per well for 10 min at room temperature, covered and in the dark. Serial dilutions of sodium nitrite (0 to 100 μM) were used to generate the nitrite standard reference curve. Following the incubation period, absorbance at 543 nm was measured using the Synergy4 microplate reader.

### Western blot analysis

Cells were harvested in lysis radioimmunoprecipitation assay (RIPA) buffer containing 50 mM Tris–HCl (pH 7.5), 150 mM NaCl, 1% Nonidet P-40, 0.5% sodium deoxycholate, and 0.1% sodium dodecyl sulfate (SDS). The extract was centrifuged at 10,000*g* for 15 min at 4°C to remove cell debris. Protein concentration was determined with the BCA protein assay kit (Pierce Biotechnology, Rockford, IL). For each sample, 5 μg of protein was loaded and resolved in 8% SDS-PAGE and run at 100 V. After electrophoresis, proteins were transferred to 0.45 μm nitrocellulose membranes at 300 mA for 3 h. Membranes were blocked in Tris-buffered saline (TBS), pH 7.4, with 0.1% Tween 20 (TBS-T) containing 5% nonfat milk for 1 h at room temperature. For different experiments, the blots were incubated with ERK1/2 (1:2000), phospho-ERK1/2 (1:2000), iNOS polyclonal (1:1000) antibodies overnight at 4°C. After repeated washing with TBS-T, blots were incubated with goat anti-rabbit IgG- horseradish peroxidase (1:4000) or goat anti-mouse IgG- horseradish peroxidase (1:2000) for 1 h at room temperature. The blots were then washed three times with TBS-T. Immunolabeling was detected by chemiluminescence ECL/WestPico/femto. For loading control, blots were incubated with monoclonal anti-β-actin peroxidase (1:30,000). For quantification, blots were scanned and the intensity of protein bands was measured as optical density using the QuantityOne program (BioRad, Hercules, CA).

### Immunostaining of p47phox and gp91phox in microglial cells

BV-2 microglia cells were serum-starved for 4 h, followed by stimulation with IFNγ (10 ng/ml) for 12 h. The cells were fixed in 4% paraformaldehyde for 15 min and then permeabilized with 0.1 % Triton X-100 in PBS for 30 min. Cells were incubated with 10% normal goat serum in 0.005% Triton X-100 in PBS for 60 min. Cells were then incubated overnight in 0.5% normal goat serum in 0.005% Triton X-100 in PBS containing primary antibodies: rabbit anti-p47phox antibodies (1:500; Calbiochem) and mouse anti-gp91phox antibodies (1:500; Thermo Fisher). The next day, cells were incubated in 0.005% Triton X-100 in PBS containing secondary antibodies, goat-anti-rabbit Alexa fluor 488 (Jackson Immunoresearch) and goat-anti-mouse Alexa fluor 549 (Jackson Immunoresearch) for 60 min, followed by nuclear counterstaining in PBS containing 1 μg/ml of 4,6-diamidine-2-phenylindole dihydrochloride (Pierce) for 10 min. The coverslips were then mounted on fluoromount (Sigma-Aldrich) and sealed with nail polish. Fluorescence photomicrographs were captured using a Leica DMI 6000B fully automated epifluorescence microscope (Leica Microsystems Inc., Buffalo Grove, IL) as serial *z*-stack images and processed for deconvolution with AF6000 applications.

### Statistical analysis

Data are presented as mean ± standard error of the mean (SEM). Results were analyzed by one-way analysis of variance (ANOVA) followed by Dunnett’s multiple comparison tests or two-way ANOVA with Bonferroni posttests (V4.00; GraphPad Prism Software Inc., San Diego, CA). Statistical significance was considered for *P* < 0.05.

## Results

### Hon and Mag suppressed NMDA-induced superoxide production in neurons

**Figure 2:**
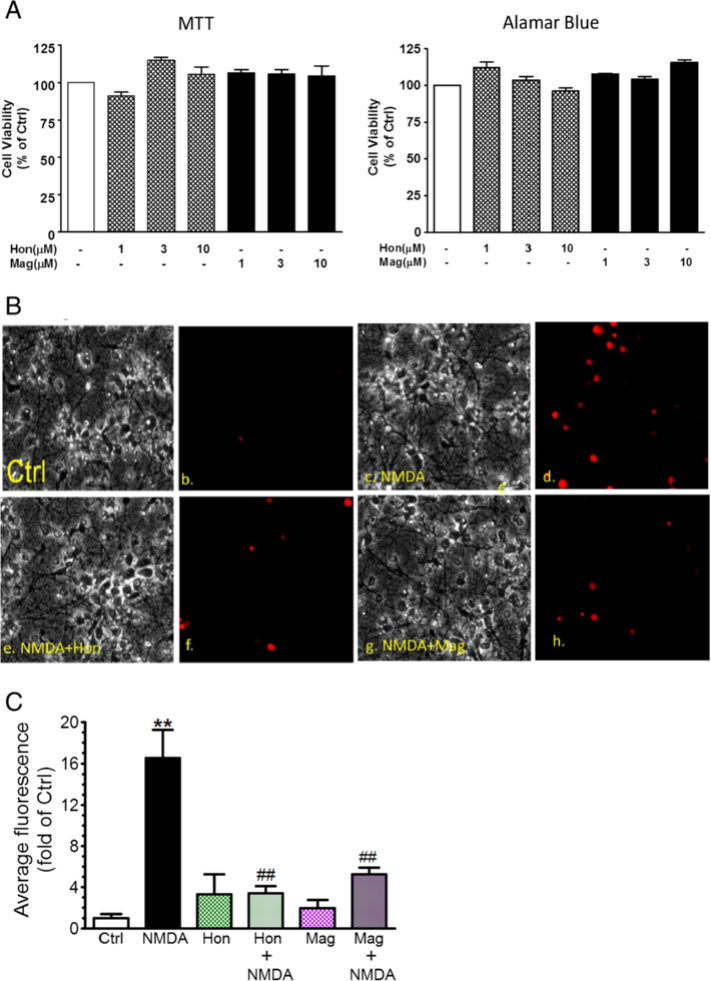
**Hon and Mag do not alter cell viability and inhibit NMDA-induced ROS production in primary rat cortical neurons. (A)** Exposure of Hon and Mag (1 to 10 μM) to primary cortical neurons for 24 h did not alter neuronal viability as assayed by MTT and AlamarBlue. **(B)** Representative bright field and DHE fluorescence photomicrographs depicting ROS production in primary neurons after cells were treated with Hon or Mag (10 μM) for 30 min prior to stimulation with NMDA (100 μM) for 30 min. For ROS production, neurons were loaded with dihydroethidium (DHE, 10 μM) 30 min prior to image acquisition under different treatment conditions. Procedure for fluorescence determination is described in text. **(C)** Bar graph of average fluorescence depicting significant inhibition of NMDA-induced ROS production by Hon and Mag. Data are expressed as the mean ± SEM from three individual experiments and analyzed by two-way ANOVA with Bonferroni posttests. ** denote significant difference between control and NMDA (*P* < 0.001), ## indicate significant decrease in ROS production by Hon and Mag as compared to NMDA (*P* < 0.01).

Since our earlier study had demonstrated the involvement of NADPH oxidase in the production of superoxide on stimulating neurons with the ionotropic glutamate receptor agonist, NMDA [16], this protocol was used to examine the ability of Hon and Mag to suppress neuronal oxidative events induced by NMDA. The MTT assay was used to mark mitochondrial dysfunction and the AlamarBlue™ assay was used to assess neuron cell death. As shown in Figure 2A, treatment of neurons with Hon and Mag (1 to 10 μM) for 24 h neither alters mitochondrial function nor causes neuron cell death.

Subsequently, we tested the effects of Hon and Mag on superoxide production in neurons stimulated with NMDA (100 μM for 30 min). Treatment of neurons with NMDA elicited a large increase in superoxide production (Figure 2B, C). Hon and Mag (10 μM) alone did not elicit superoxide production in neurons; however, both compounds significantly inhibited NMDA-induced superoxide production (Figure 2B, C).

### Hon and Mag inhibited IFNγ- and LPS-induced iNOS expression and NO production in BV-2 microglial cells

**Figure 3:**
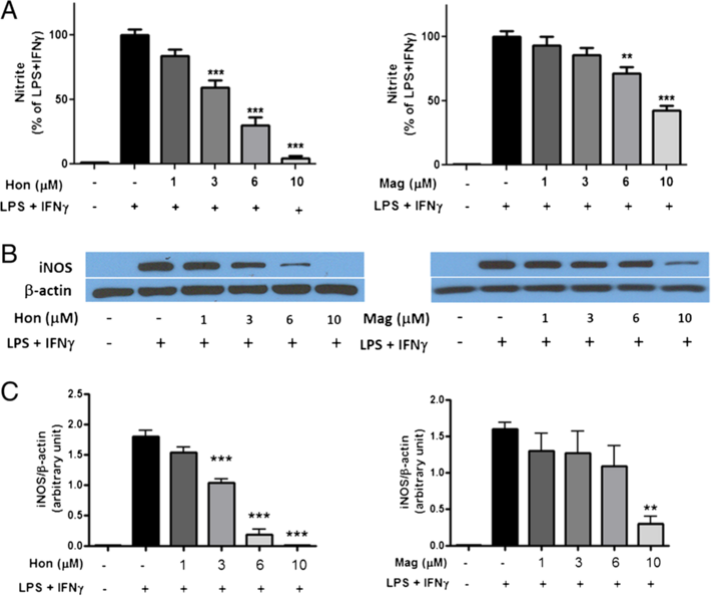
**Hon and Mag inhibit LPS + IFNγ-induced NO production and iNOS expression in BV-2 microglial cells. **Cells were treated with Hon or Mag (1 to 10 μM) for 1 h followed by stimulation with LPS (100 ng/ml) + IFNγ (10 ng/ml) for 16 h. **(A)** Culture media were collected for determination of NO using the Griess reaction protocol as described in text. **(B)** Representative Western blots of iNOS protein and β-actin. **(C)** Bar graphs representing iNOS/β-actin ratios. Results were expressed as the mean ± SEM (*n* = 3) and significant difference from the respective LPS + IFNγ stimulated group was determined by one-way ANOVA followed by Dunnett’s tests, ** *P* < 0.01; ***P < 0.001.

As with our previous study [25], treatment of BV-2 microglial cells with IFNγ + LPS caused the induction of iNOS expression and a correlative increase in NO production (Figure 3). Hon and, to a lesser extent, Mag inhibited LPS+IFNγ-induced iNOS expression and NO production in BV-2 cells in a dose-dependent manner (Figure 3). Significant inhibition of NO (*P* < 0.05) by Hon was observed at 1 μM, whereas significant inhibition for Mag was observed at 6 μM. Western blot analysis also indicated a dose-dependent inhibition of iNOS protein expression by Hon and Mag (Figure 3B, C).

**Figure 4:**
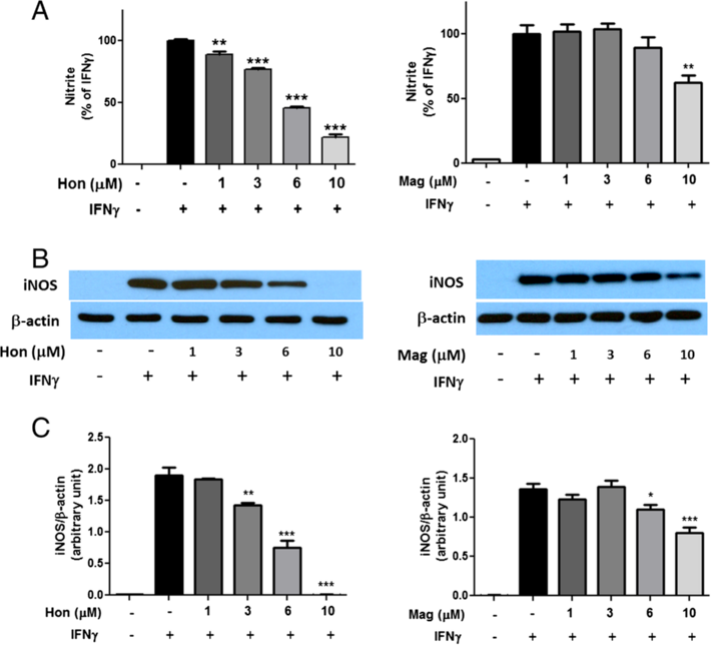
**Hon and Mag inhibit IFNγ-induced NO production and iNOS expression in BV-2 microglial cells. **Cells were treated with Hon or Mag (1 to 10 μM) for 1 h followed by stimulation with IFNγ (10 ng/ml) for 16 h. **(A)** Culture media were collected for determination of NO using the Griess reaction protocol as described in text. **(B)** Representative Western blots of iNOS protein and β-actin. **(C)** Bar graphs representing iNOS/β-actin ratios. Results were expressed as the mean ± SEM (*n* = 3) and significant difference from the respective IFNγ stimulated group was determined by one-way ANOVA followed by Dunnett’s tests, *P < 0.05, **P < 0.01, ***P < 0.001.

Similar to results observed by our earlier study [25], addition of IFNγ to BV-2 microglial cells greatly stimulated LPS-induced iNOS expression and NO production. Under this condition, Hon and, to a lesser extent, Mag could dose-dependently inhibit the induction of iNOS and NO induced by IFNγ (Figure 4A-C). In fact, inhibition of IFNγ-induced NO production was similar to that using LPS + IFNγ (Figure 3A).

**Figure 5:**
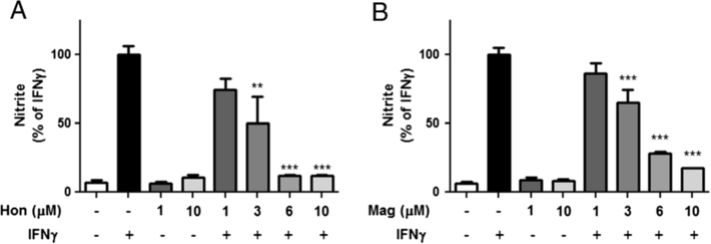
**Hon and Mag inhibit IFNγ-induced NO production in HAPI microglial cells. **Cells were treated with **(A)** Hon or **(B)** Mag (1 to 10 μM) for 1 h followed by stimulation with IFNγ (10 ng/ml) for 16 h. Culture media were collected for determination of NO using the Griess reaction protocol as described in text. Results were expressed as the mean ± SEM (*n* = 3) and significant difference from the respective IFNγ-stimulated group was determined by one-way ANOVA followed by Dunnett’s tests, *P < 0.05, **P < 0.01, ***P < 0.001.

To confirm the effects of Hon and Mag on IFNγ-induced NO production in other types of microglial cells, rat HAPI microglial cells were tested. As shown in Figure 5A, similar results were obtained for Hon to inhibit IFNγ-induced NO in HAPI cells. On the other hand, Mag appeared to provide greater inhibition in HAPI cells as compared to BV-2 cells (Figure 5B).

### ROS production in BV-2 microglial cells

**Figure 6:**
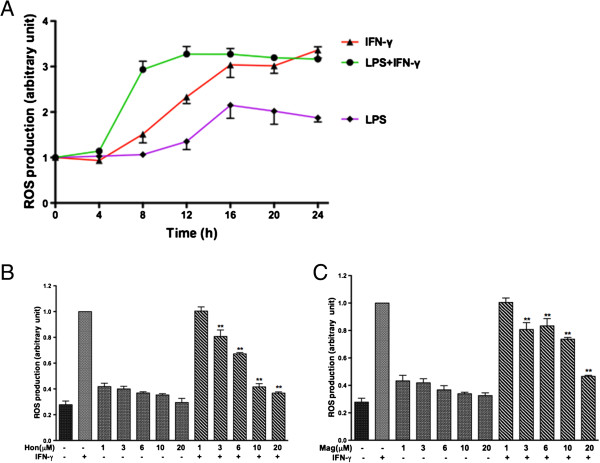
**Hon and Mag inhibit cytokine-induced ROS production in BV-2 microglial cells. (A)** Time course for ROS production induced by LPS or IFNγ. Cells were treated with LPS (100 ng/ml) or IFNγ (10 ng/ml) for the time indicated. ROS production was measured using CM-H2DCFDA as described in the text. Similarly, **(B)** Hon and **(C)** Mag (1 to 10 μM) were given 1 h prior to exposure to IFNγ (10 ng/ml) for 12 h. Results are expressed as the mean ± SEM (*n* = 3) and significant difference from the respective IFNγ-stimulated group was determined by one-way ANOVA followed by Dunnett’s tests, *P < 0.05; ** *P* < 0.01.

Although previous studies have demonstrated the ability for LPS + IFNγ to induce ROS production in microglial cells, little is known about the source and the mechanism of action of the cytokines. In this study, a time course experiment was carried out to examine ROS production on treating BV-2 microglial cells to LPS or IFNγ. Data in Figure 6A showed no detectable ROS during the initial 4 h after exposing cells to LPS or IFNγ. Lipopolysaccharide alone showed a slow increase in ROS after 4 h and peaked at 16 h, whereas IFNγ induced about 50% more ROS than LPS. When cells were treated with IFNγ together with LPS, there was a left shift for ROS production although the peak level was similar to that induced by IFNγ alone (Figure 6A).

Based on the time course conditions, we used the 12 h time point to examine whether Hon and Mag may alter IFNγ-induced ROS production. Results indicated that Hon and, less effectively, Mag did inhibit IFNγ-induced ROS production in a dose-dependent manner. However, neither compound alone could exert obvious effects on the basal ROS production (Figure 6B, C).

### NADPH oxidase is involved in IFNγ-induced ROS production

**Figure 7:**
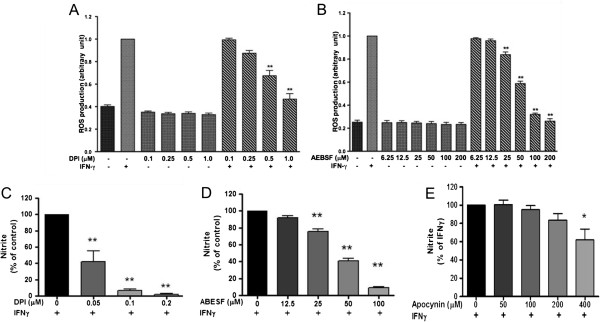
**Activation of NADPH oxidase and ROS production is upstream of NO production in BV-2 microglial cells. **Cells were pretreated with **(A)** DPI (0.1 to 1.0 μM) or **(B)** AEBSF (6.25 to 200 μM) for 1 h prior to exposure to IFNγ (10 ng/ml) for 12 h, followed by measurement of ROS with CM-H2DCFDA as described in text. Cells were pretreated with **(C)** DPI (0.05 to 0.2 μM), **(D)** AEBSF (12.5 to 100 μM), or **(E)** apocynin (50 to 400 μM) for 1 h prior to exposure to IFNγ (10 ng/ml) for 16 h, followed by measure of nitrite with Griess reaction protocol as described in the text. Results are expressed as the mean ± SEM (*n* = 3), and significant difference from the respective IFNγ-stimulated group was determined by one-way ANOVA followed by Dunnett’s tests, *P < 0.05; **P < 0.01.

We further investigated whether NADPH oxidase might be the target enzyme for the ROS induced by IFNγ in microglial cells by testing with inhibitors commonly used for NADPH oxidase. Results show that DPI, a nonspecific oxidase inhibitor, and AEBSF, a serine protease inhibitor [26], could inhibit IFNγ-induced ROS in microglial cells in a dose-dependent manner (Figure 7A, B).

Using these inhibitors, we further tested whether IFNγ-induced ROS is critical for downstream signaling pathway leading to NO production. As shown in Figure 7C and D, DPI and AEBSF similarly inhibited IFNγ-induced NO production. Apocynin, a botanical compound known to inhibit translocation of cytosolic subunits p47phox and p67phox to the membrane complex [27], was shown to inhibit IFNγ-induced NO production albeit at high concentrations, starting at 40 μM (Figure 7E).

**Figure 8:**
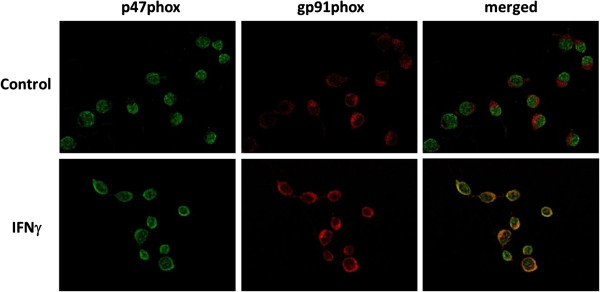
**Involvement of NADPH oxidase in IFNγ-induced NO production in BV-2 microglial cells. **Cells were exposed to IFNγ (10 ng/ml) for 12 h. Immunocytochemistry was performed with antibodies against p47phox (green) and gp91phox (red). Images were taken under fluorescence microscope as serial *z*-stack images, followed by deconvolution processing.

Activation of NADPH oxidase is known to act through a complex mechanism involving activation of cytosolic subunits (p47phox, p67phox, p40phox, Rac) to dock with the membrane subunits (gp91phox, p22phox). In the past, several types of kinase have been shown to phosphorylate the cytosolic subunits (p47phox, p67phox, p40phox) [28]. In this study, double immunocytochemical staining was used to demonstrate that IFNγ could induce the translocation of p47phox from the cytosol to dock with the gp91phox in the membrane (Figure 8), further supporting the involvement of the NADPH oxidase in the ROS production.

### IFNγ-induced phosphorylation of MAPK/ERK1/2

**Figure 9:**
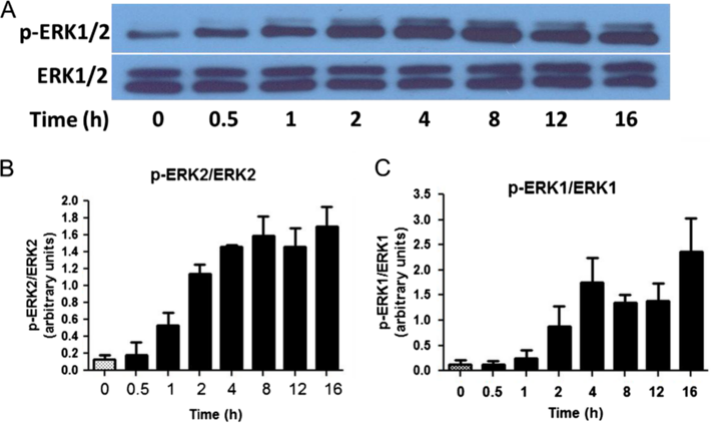
**Time course of IFNγ-induced activation of p-ERK1/2 in BV-2 microglial cells. (A)** Western blot analysis of a typical time course for IFNγ (10 ng/ml) to induce ERK1/2 phosphorylation in BV-2 microglia cells. Cell lysates were extracted at the time indicated. **(B, C)** Results of protein band intensities are expressed as arbitrary units of phospho-ERK1/2 against total ERK1/2 for **(B)** ERK1 and **(C)** ERK2. Results are expressed as mean ± SEM (*n* = 3).

**Figure 10:**
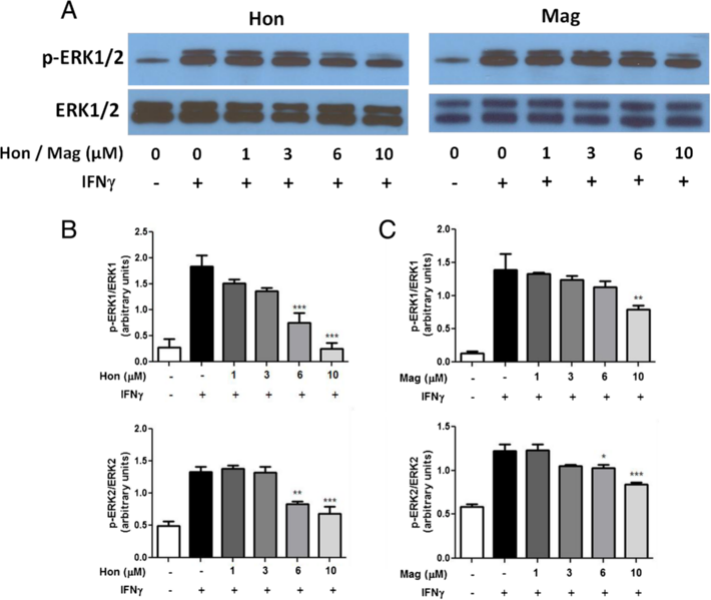
**Hon and Mag inhibit IFNγ-induced activation of p-ERK1/2 in BV-2 microglial cells. (A)** Western blot analysis showing a representative experiment of Hon or Mag pretreatment on IFNγ to induce p-ERK1/2 phosphorylation in BV-2 microglia cells. Cells were treated with either Hon or Mag (1 to 10 μM) for 1 h followed by stimulation with IFNγ (10 ng/ml) for 4 h. **(B** &**C)** Results of protein band intensities are expressed as arbitrary units of phospho-ERK1/2 against total ERK1/2. Results are expressed as the mean ± SEM (*n* = 3) and significant difference from the respective IFNγ stimulated group was determined by one-way ANOVA followed by Dunnett’s tests, *P < 0.05; ** *P* < 0.01; *** *P* < 0.001.

**Figure 11:**
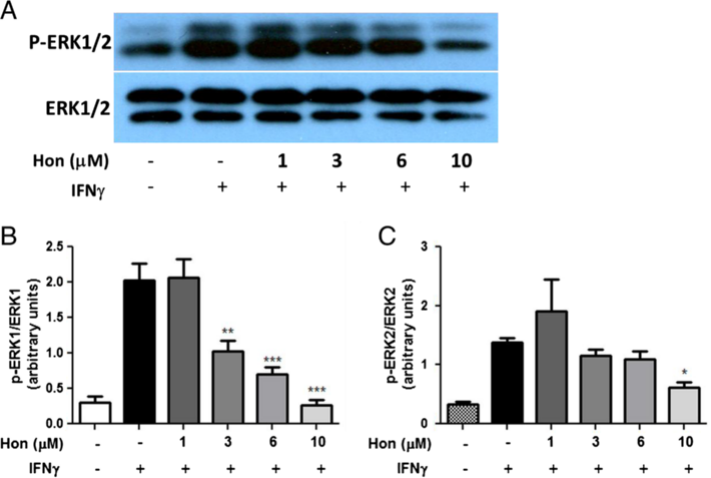
**Hon inhibits IFNγ-induced activation of p-ERK1/2 in HAPI microglial cells. (A)** Western blot analysis showing a representative experiment of Hon pretreatment on IFNγ to induce ERK1/2 phosphorylation in HAPI microglial cells. Cells were treated with Hon (1 to 10 μM) for 1 h followed by stimulation with IFNγ (10 ng/ml) for 4 h. **(B** &**C)** Results of protein band intensities are expressed as arbitrary units of phospho-ERK1/2 against total ERK1/2. Results are expressed as the mean ± SEM (*n* = 3) and significant difference from the respective IFNγ stimulated group was determined by one-way ANOVA followed by Dunnett’s tests, *P < 0.05; ** *P* < 0.01; *** *P* < 0.001.

Although cytokines and LPS were shown to induce phosphorylation of ERK1/2, it is not clear whether this response is a reflection of IFNγ [25]. Therefore, a time course assay was carried out to examine the ability of IFNγ to induce p-ERK1/2. IFNγ induced a steady increase in p-ERK1/2 expression, which was noticeable around 1 h and reached a plateau after 4 h (Figure 9A-C). Hon and, to a lesser extent, Mag inhibited IFNγ-induced ERK1/2 phosphorylation in a dose-dependent manner (Figure 10A-C). Similarly, Hon also inhibited IFNγ-induced pERK1/2 in HAPI cells (Figure 11A-C).

### IFNγ-induced increase in p-ERK1/2 is upstream of ROS and NO production

**Figure 12:**
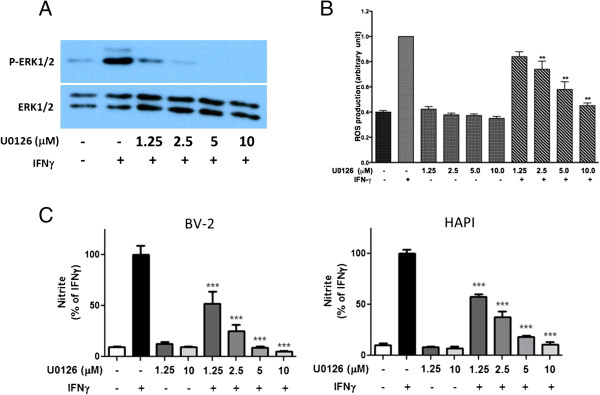
**Role of ERK1/2 activation in IFNγ-induced ROS and NO production in BV-2 and HAPI microglial cells. **Cells were treated with IFNγ (10 ng/ml) with or without the MEK1/2 inhibitor, U0126 (1.25 to 10 μM). **(A)** Representative Western blot demonstrated ability for U0126 to inhibit phosphorylation of ERK1/2 dose-dependently 4 h after IFNγ treatment. **(B)** For ROS production, BV-2 cells were pretreated with different concentrations of U0126 for 1 h prior to stimulation with IFNγ for 12 h. **(C)** For NO production, both BV-2 and HAPI cells were pretreated with different concentrations of U0126 for 1 h prior to stimulation with IFNγ for 16 h. Results are expressed as the mean ± SEM (*n* = 3), and significant difference from the respective IFNγ stimulated group was determined by one-way ANOVA followed by Dunnett’s tests, ** *P* < 0.01; *** *P* < 0.001.

To test whether IFNγ-induced p-ERK1/2 is upstream of ROS and NO production, the MEK1/2 inhibitor, U0126, was used to block phosphorylation of ERK1/2 by MEK1/2. As shown in Figure 12A, U0126 effectively inhibited IFNγ-induced p-ERK1/2 in BV-2 cells. U0126 also inhibited IFNγ-induced ROS production in a dose-dependent manner, whereas U0126 did not influence basal ROS production in BV-2 cells (Figure 12B). Under similar conditions, U0126 was shown to inhibit IFNγ-induced NO production in BV-2 and HAPI cells (Figure 12C).

## Discussion

In this study, we used rat primary cortical neurons and immortalized microglial cells (murine BV-2 and rat HAPI) to demonstrate the ability of Hon and Mag to suppress oxidative and inflammatory responses induced by NMDA in neurons and IFNγ ± LPS in microglial cells. Exposure of neurons to Hon and Mag (up to 10 μM) for 24 h did not alter neuronal viability, but both compounds (10 μM) dramatically inhibited NMDA-induced superoxide production. Results from earlier studies have demonstrated involvement of NADPH oxidase in neuronal excitation [16,17], and Mag and Hon were shown to offer protection against neuronal toxicity induced by hydrogen peroxide and ionotropic glutamate receptor agonist [29]. These and other results are in line with the suggestion of possible therapeutic application of these compounds to treat neurological disorders [1].

The induction of iNOS expression and NO production by pro-inflammatory cytokines and LPS is known to involve the transcriptional pathways associated with NF-κB and the canonical JAK/STAT pathway induced by IFNγ [22,30]. Interestingly, in BV-2 and HAPI microglial cells, LPS and IFNγ can individually stimulate iNOS/NO production, suggesting presence of cross-talk mechanisms between these two pathways [25,31,32]. There is evidence that signaling molecules, such as protein kinase C and mitogen-activated protein kinase (MAPK), can mediate the cross-talk pathways [22,33]. In studies with microglial cells, increase in MAPK-ERK1/2 activity has been shown to occur during induction of iNOS by LPS and IFNγ [34-36]. Our results here also demonstrated the ability for IFNγ alone to stimulate phosphorylation of ERK1/2 in microglial cells (Figure 9A, B). However, unlike studies in which neurons show a rapid induction of p-ERK1/2 in minutes in response to NMDA stimulation [16], phosphorylation of ERK1/2 by IFNγ in BV-2 microglial cells turned out to be a much slower and gradual process, first observable after 1 h and reaching a plateau around 4 h. In human macrophages, stimulation of p-ERK1/2 by IFNγ exhibited an even longer delay time, of 6 h [37]. Apparently, the time required for ERK1/2 phosphorylation varies depending on the cell types and agonists used for activation [38].

Although pro-inflammatory cytokines have been shown to stimulate ROS production in microglial cells, the mechanisms for ROS production have not been clearly elucidated [39]. In our study with microglial cells, ROS production induced by LPS and IFNγ was not observed before 4 h (Figure 6A). Since IFNγ-induced ROS production was inhibited by the MEK inhibitor (U0126, Figure 12B), it is reasonable to conclude that p-ERK1/2 plays an important role in the induction process. Our results support earlier studies demonstrating the involvement of ERK1/2 from IFNγ to stimulate superoxide in a mixed glial cell culture [36]. Our study further provided evidence for involvement of NADPH oxidase in IFNγ-induced ROS production in microglial cells. Inhibitors, such as DPI and AEBSF, not only inhibited ROS but also NO production, suggesting that ROS production precedes induction of iNOS. To demonstrate further the involvement of NADPH oxidase in IFNγ-induced ROS production, double immunocytochemical staining showed translocation of p47phox from cytoplasm to membrane and co-localized with the gp91phox after treating cells with IFNγ (Figure 8). In a study with macrophages, IFNγ was shown to activate NADPH oxidase through increase in intracellular trafficking and expression of gp91phox [40].

Many extracellular signals are coupled with cell surface receptors linking to activation of the MAPK pathway. In this study, we demonstrated the role of IFNγ in stimulating ROS from NADPH oxidase through the MEK-ERK1/2 pathway. Indeed, activation of ERK1/2 has been shown to elicit multiple downstream events, including phosphorylation of cytosolic phospholipase A2 (cPLA2) and arachidonic acid release in neurons [16], production of filopodia in BV-2 microglial cells [25], transcytosis of macromolecules across the epithelial monolayers [37], and enhanced phosphorylation of STAT1 [41].

Taken together, these results demonstrated a novel role of IFNγ in stimulating the MAPK-ERK1/2 pathway, and in turn, in activating ROS production through NADPH oxidase. Since IFNγ-induced p-ERK is upstream of ROS and iNOS/NO production, agents that inhibit p-ERK1/2 can shut down both ROS production and iNOS induction. Understanding this mechanism helps to explain earlier observations that LPS + IFNγ induced ROS production in microglial cells preceded the induction of iNOS [42].

Many natural polyphenols, including luteolin, gastrodin, and ginsenoside, exhibit anti-inflammatory properties and inhibit LPS/IFNγ-induced NO production in microglial cells, either through ERK1/2 or other types of MAPK [7,43-47]. Other studies have demonstrated the ability of Hon and Mag to inhibit cytokine-induced NO production and expression of iNOS, as well as generation of prostaglandins and leukotrienes [18-20,48]. Our studies unveiled the role of the IFNγ-ERK1/2 pathway in ROS production through NADPH oxidase, and the ability of Hon and Mag to suppress this oxidative and inflammatory responses in microglial cells. Understanding this mechanism can help to explain the ability of these botanicals to suppress hyperactivity in brain and retard microglial cell activation and ameliorate neuroinflammatory responses associated with neurodegenerative diseases.

## Conclusion

In summary, our studies with neurons and microglial cells provided strong evidence for anti-oxidative and anti-inflammatory effects of Hon and Mag and underscore the important role of NADPH oxidase in the superoxide/ROS production in these cells. Results with microglial cells further unveiled the important role of IFNγ in stimulating signaling pathways involving activation of ERK1/2, ROS, and NO. These results also provide a useful and novel platform for testing anti-oxidative and anti-inflammatory effects of other botanicals.

## Abbreviations

AEBSF: 4-(2-aminoethyl)-benzenesulfonylfluoride; ANOVA: Analysis of variance; BSA: Bovine serum albumen; CCD: Charge-coupled device; DCF: CM-H2DCFDA; DHE: Dihydroethidium; DMEM: Dulbecco’s modified Eagle’s medium; DMSO: Dimethyl sulfoxide; DPI: Diphenyleneiodonium; FBS: Fetal bovine serum; HPLC: High performance liquid chromatography; IFNγ: Interferon-γ; iNOS: Inducible nitric oxide synthase; LPS: Lipopolysaccharide; MAPK: Mitogen-activated protein kinase; MTT: Methylthiazolyldiphenyl-tetrazolium bromide; NIH: National Institutes of Health; NMDA: N-methyl-D-aspartate; NO: Nitric oxide; PBS: Phosphate-buffered saline; RIPA: Radioimmunoprecipitation assay; ROS: Reactive oxygen species; SDS: Sodium dodecyl sulfate; SEM: Standard error of the mean; TBS: Tris-buffered saline.

## Competing interests

The authors do not have competing interests.

## Authors’ contributions

DYC carried out the studies to elucidate IFNγ-ERK1/2 pathway and immunocytochemistry, MHC provided the botanicals and carried out initial studies to test Hon and Mag on cytokine-induced iNOS while visiting the University of Missouri, YZ contributed to studies on ROS in microglial cells and the elucidation of NADPH oxidase, WS carried out initial cell culture studies, YH participated in studies with neurons, JHJ carried out studies to test the effects of Hon and Mag on NADPH oxidase, JC assisted in preparation of the primary neurons, JCL contributed the protocol for assay of ROS in neurons, AS participated in the design of the study and performed the statistical analysis. While GYS is the leader of this project, DYC, MHC, AS, ZG, KLF, WRF, DBL, and AYS participated in discussions of concepts, and coordinated and helped in drafting the manuscript. All authors have read and approved the final manuscript.

## Authors’ information

This work is dedicated to Dr. Albert Y. Sun, who devoted his life to the study of polyphenols in health and diseases. He passed away on 30 April 2012.
